# Defining a Subgroup Treatable for Laparoscopic Surgery in Poorly Differentiated Early Gastric Cancer: the Role of Lymph Node Metastasis

**DOI:** 10.3969/j.issn.2095-3941.2012.01.010

**Published:** 2012-03

**Authors:** Zhi-bin Huo, Shuo-po Chen, Hua Li, Dian-chao Wu

**Affiliations:** 1 Department of Surgical Oncology;; 2 Department of Surgical Urology, Affiliated Xingtai People Hospital of Hebei Medial University, Xingtai 054001, China

**Keywords:** gastric cancer, lymph nodes, metastasis, laparoscopy

## Abstract

**Objective:**

The present study aims to identify the clinicopathologic factors predictive of lymph node metastasis (LNM) in poorly differentiated early gastric cancer (EGC) and to expand the possibility of using laparoscopic surgery for the treatment of poorly differentiated EGC.

**Methods:**

Data from 70 cases of poorly differentiated EGC treated with surgery were collected. The association between clinicopathologic factors and the presence of LNM was retrospectively analyzed by univariate and multivariate logistic regression analyses.

**Results:**

Univariate analysis showed that tumor size, depth of invasion, and lymphatic vessel involvement (LVI) were the significant and independent risk factors for LNM (all *P*<0.05). The LNM rates were 6.9%, 45.5%, and 60.0%, respectively. There was no LNM in 25 patients without the above three risk factors.

**Conclusions:**

Laparoscopic surgery is a sufficient treatment for intramucosal poorly differentiated EGC if the tumor is less than or equal to 2.0 cm in size and when LVI is absent upon postoperative histological examination.

## Introduction

Early gastric cancer (EGC) is defined as gastric carcinoma in which the invasion is confined to the mucosa or submucosa regardless of the presence of lymph node metastasis. The minimalization of therapeutic invasiveness to preserve quality of life is a major topic in the management of EGC. After laparoscopic surgery for gastric cancer was introduced by Kitano et al. ^[^^1^^]^ in 1994, the development of laparoscopic procedures has been aggressively pursued. This minimally invasive technique can be applied to manage EGC without the risk of lymph node metastases (LNM) ^[^^2^^-^^5^^]^. The application of laparoscopic surgery has been limited to differentiated EGC because of the higher risk of lymph node metastases in poorly differentiated EGC compared with differentiated EGC ^[^^6^^]^. Therefore, gastrectomy with lymphadenectomy has been considered as an essential treatment for patients with poorly differentiated EGC. However, almost all (96.6%) surgical cases of poorly differentiated EGC confined to the mucosa have been found not to have LNM ^[^^7^^]^, suggesting that gastrectomy with lymphadenectomy may be an overtreatment for these cases.

Therefore, this retrospective study aims to determine the clinicopathologic factors that are predictive of LNM in poorly differentiated EGC, and to establish a simple criterion to expand the possibility of using laparoscopic surgery for the treatment of poorly differentiated EGC.

## Patients and Methods

### Patients

Patients who underwent radical operation due to EGC in the Department of Surgical Oncology, Affiliated Xingtai People Hospital of Hebei Medical University, Xingtai, China between January 1994 and December 2009 were included in the screening for identification of cases with EGC in this retrospective study.

The inclusion criteria for the study were as follows: (1) lymph node dissection beyond limited (D1) dissection was performed; (2) the resected specimens and lymph nodes were pathologically analyzed, and poorly differentiated EGC was diagnosed according to the Japanese Classification of Gastric Carcinoma (JCGC) ^[^^8^^]^; and (3) the patient’s medical record was available in the database.

During the previous 15 years, 70 patients (49 males, 21 females; mean age of 50 years, ranged from 29 to 80 years) with histopathologically poorly differentiated EGC were identified to meet the inclusion criteria for further analysis in this study.

### Dissection of lymph nodes and assessment and classification of lymph node metastasis

The lymph nodes of each case were meticulously dissected from enbloc specimens, and their classification was determined by a surgeon based on JCGC after a careful review of the excised specimens ^[^^7^^]^. Then the resected lymph nodes were sectioned and stained with hematoxylin and eosin and examined by pathologists for metastasis and lymphatic vessel involvement (LVI).

### Association between clinicopathologic parameters and lymph node metastasis

The following clinicopathologic parameters that are covered in JCGC ^[^^8^^]^ were included in this study: gender (male and female), age (<60 years, ≥60 years), family medical history of gastric cancer, number of tumors (single or multitude), location of the tumor (upper, middle, or lower part of the stomach), tumor size (maximum dimension ≤2 cm, or >2 cm), macroscopic type [protruded (type I), superficial elevated (type IIa), flat (type IIb), superficial depressed (type IIc), or excavated (type III)], depth of invasion (mucosa, submucosa), and lymphatic vessel involvement. The associations between various clinicopathologic factors and LNM were examined as described below.

### Statistical analysis

All data were analyzed using SPSS15.0 statistical software (Chicago, IL United States). The differences in clinicopathologic parameters between patients with and without LNM were determined by χ^2^ test. A multivariate stepwise logistic regression analysis was subsequently performed to identify the independent risk factors for LNM. The hazard ratio and 95% confidence interval (CI) were calculated. A *P* value of less than 0.05 was considered statistically significant.

## Results

### Association between clinicopathologic factors and lymph node metastasis

The association between various clinicopathologic factors and LNM was first analyzed by χ^2^ test (**Table 1**). A tumor larger than 2.0 cm, submucosal invasion, and the presence of LVI were significantly associated with a higher rate of LNM (all *P*<0.05). However, gender, age, family history of gastric cancer, number, location, and macroscopic type were not associated with LNM.

**Table 1:** Univariate analysis of potential risk characteristics for lymph node metastasis.

Characteristics	Lymph node metastasis positive number (%)	*P*
Gender		
Male (*n*=49)	6 (12.2)	0.523
Female (*n*=21)	4 (19.0)	
Age, years		
<60 (*n*=65)	9 (13.8)	0.748
≥60 (*n*=5)	1 (20.0)	
Family history		
Positive (*n*=1)	0	0.704
Negative (*n*=69)	10 (14.5)	
Number of tumors		
Single (*n*=68)	10 (14.7)	0.588
Multitude (*n*=2)	0	
Location		
Upper (*n*=16)	3 (20.0)	
Middle (*n*=4)	0	0.683
Lower (*n*=50)	7 (14.0)	
Tumor size, cm		
≤2 (*n*=45)	2 (4.4)	0.008
>2 (*n*=25)	8 (32.0)	
Macroscopic type		
I (*n*=0)	0	
II (*n*=55)	8 (13.6)	0.171
III (*n*=15)	2 (40.0)	
Depth of invasion		
Mucosa (*n*=46)	2 (4.3)	0.006
Submucosa (*n*=24)	8 (33.3)	
Lymphatic vessel involvement		
Positive (*n*=11)	6 (54.5)	0.001
Negative (*n*=59)	4 (6.8)	

### Multivariate analysis of potential independent risk clinicopathological factors for lymph node metastasis

The three characteristics significantly associated with LNM based on univariate analysis were found to be significant and independent risk factors for LNM based on multivariate analysis (both *P*<0.05, **Table 2**).

**Table 2:** Multivariate analysis of potential risk factors for lymph node metastasis.

Characteristics	HR	95%CI	*P*
Tumor size, cm			
≤2 (*n*=45)	6.235	1.150-35.112	0.038
>2 (*n*=25)			
Depth of invasion			
Mucosa (*n*=46)	13.354	1.371-97.014	0.031
Submucosa (*n*=24)			
Lymphatic vessel involvement			
Positive (*n*=11)	32.012	1.657-185.397	0.017
Negative (*n*=59)			

### Lymph node metastasis in poorly differentiated EGC

LNM was histologically confirmed in 10 (14.3%) of the 70 patients. The LNM rates were 6.9%, 45.5%, and 60% in cases with one, two, and three of the risk factors in poorly differentiated EGC, respectively. There was no LNM in 25 patients without the three risk factors (**Table 3**).

**Table 3:** Relationship between the number of risk factors and lymph node metastasis in poorly differentiated EGC.

No. of positive risk factors	Nodal metastasis rate, % (*n*)
None	0 (0/25)
One	6.9 (2/29)
Two	45.5 (5/11)
Three	60.0 (3/5)

## Discussion

Early gastric cancer (EGC) was defined in 1963 as adenocarcinoma confined to the mucosa or submucosa irrespective of lymph node involvement. This definition has been widely accepted and excellent outcomes have been reported from both the West and the East. Given the increased rate of the accurate diagnosis of EGC, which in turn leads to improved prognosis, increased interest has been focused on minimization of invasive procedures and the improvement of quality of life.

Laparoscopic surgery for EGC was first reported by Kitano et al.^[^^1^^]^ in 1994. A survey by the Japan Society for Endoscopic Surgery showed satisfactory results on morbidity and mortality in the early period, and today laparoscopic surgery is being performed as an optional treatment for EGC at many institutions in Japan and Korea. Laparoscopic surgery has been associated with less pain, faster recovery of gastrointestinal function, better pulmonary function, decreased stress response, shorter hospital stay, and better postoperative quality of life than open gastrectomy ^[^^9^^, ^^10^^]^.

The present multivariate analysis revealed that a tumor larger than 2.0 cm, submucosal invasion, and the presence of LVI were significant predictive factors for LNM in patients with poorly differentiated EGC. These results corroborated those of previous reports on undifferentiated EGC, which demonstrated a significant correlation between the high incidence of LNM and a tumor larger than 2.0 cm, submucosal invasion, or presence of LVI ^[^^11^^, ^^12^^]^.

This study found no LNM in patients with intramucosal cancer if the tumor was less than or equal to 2.0 cm in size without LVI, possibly indicating that laparoscopic surgery could be sufficient to treat these cases and that additional surgery was unnecessary.

In the present study, the LNM rates were 6.9%, 45.5%, and 60% in cases with one, two, and three of the risk factors in poorly differentiated EGC, respectively. Therefore, gastrectomy with lymphadenectomy is inevitable for these patients with risk factors.

A small randomized study revealed several advantages of laparoscopic surgery including less pain and less impairment of pulmonary function. Many case series showed that the procedure is technically safe and brings better short-term outcomes such as faster recovery, shorter hospital stay, less pain and better cosmesis when compared to open surgery. Moreover, remarkable advances in laparoscopic surgical techniques and instruments have been achieved, such as laparoscopic coagulating shears. Extended lymph node dissection (D2) and total gastrectomy can now be performed laparoscopically.

According to this study, laparoscopic surgery alone should be a sufficient treatment for intramucosal poorly differentiated EGC if the tumor is less than or equal to 2.0 cm in size and LVI is absent upon postoperative histological examination. In the future, laparoscopic procedures for gastric cancer may be widely accepted in China if the advantages of laparoscopic approaches are confirmed in randomized controlled trials involving long-term outcomes.
